# Functional Outcomes of Anatomic Total Shoulder Arthroplasty in Patients Over 60 Years Old: A Retrospective Study (2019–2024)

**DOI:** 10.7759/cureus.100210

**Published:** 2025-12-27

**Authors:** Lina Abbakr, Joshua Nadimi, Christos Dragonas, Andreas Fontalis, Maariya Tariq, Dimitra Leivadiotou

**Affiliations:** 1 Trauma and Orthopaedics, The Princess Alexandra Hospital NHS Trust, Harlow, GBR; 2 Trauma and Orthopaedics, Royal London Hospital, London, GBR

**Keywords:** american shoulder and elbow surgeons score (ases), anatomic total shoulder arthroplasty, arthroplasty, functional, functional and clinical outcome, glenohumeral osteoarthritis, oxford shoulder score, replacement, reverse shoulder arthoplasty, shoulder

## Abstract

Background

This study aimed to evaluate the functional outcomes, complications, and revision rates of anatomic total shoulder arthroplasty (aTSA) in patients over 60 years old with intact rotator cuffs.

Methodology

A retrospective analysis was conducted among 42 (100%) patients aged over 60 who underwent aTSA between 2019 and 2024 in two hospitals. Patients were included if they had severe shoulder arthritis and an intact rotator cuff. All procedures utilised a standardised deltopectoral approach with implantation of a stemmed uncemented humeral component and a keeled cemented polyethylene glenoid (Tornier Perform Anatomic Glenoid, Stryker Corporation, Kalamazoo, MI, USA), with a minimum follow-up of 12 months postoperatively. Demographic data were collected along with pre- and postoperative Oxford Shoulder Score (OSS) and American Shoulder and Elbow Surgeons (ASES) scores, glenoid morphology (assessed via the Walch classification), and complications. Statistical analysis was performed using paired t-tests to compare pre- and postoperative scores.

Results

The study included 42 (100%) patients; 35 (83%) females and 7 (16.7%) males with a mean age of 74.5 years. The mean duration of follow-up was 30 months. The mean preoperative OSS was 18.3 (SD = 9.7), which significantly improved to 46 postoperatively (SD = 3.1, range = 38-48, p < 0.0001). Overall, 40 (95.23%) patients achieved a postoperative OSS over 40, with 20 (47.62%) patients scoring the maximum of 48. The ASES score averaged 93, with 33 (78.57%) patients scoring between 80 and 100. Glenoid morphology analysis revealed A2 as the most common type observed in 18 (42.86%) patients. Complications were minimal, with occasional pain reported in four (9.52%) patients, while no patients returned to theatre.

Conclusions

aTSA demonstrates significant improvements in shoulder function and pain relief for elderly patients with intact rotator cuffs. The high postoperative OSS and ASES scores, along with low complication rates, support the effectiveness of aTSA in this population. These findings suggest that it is a viable and successful surgical option for elderly patients with glenohumeral osteoarthritis, even in those over 70 years old, challenging the growing trend toward reverse total shoulder arthroplasty in this age group. Further research is needed to explore long-term outcomes and refine surgical indications.

## Introduction

Functional outcomes following shoulder arthroplasty are commonly assessed using validated instruments such as the Oxford Shoulder Score (OSS) [1] and the American Shoulder and Elbow Surgeons (ASES) score [2], which evaluate pain, range of motion, and daily function. With these tools, clinicians can objectively measure improvements and compare outcomes across patient populations.

The choice of the optimal surgical treatment for patients over 60 years old with glenohumeral arthritis, particularly those with an intact rotator cuff, remains a critical consideration in shoulder surgery. Anatomic total shoulder arthroplasty (aTSA) has been widely recognised as a reliable and effective option for managing end-stage glenohumeral osteoarthritis, especially in patients with preserved rotator cuff function [3-5]. Its design, which replicates the natural anatomy and function of the shoulder, allows for significant restoration of function, range of motion, and pain relief, making it a preferred choice for patients with primary osteoarthritis and a functional rotator cuff [3,4]. The modern aTSA design aims to maintain natural biomechanics of the shoulder, driven by the rotator cuff, which offers predictable outcomes and long-term durability in appropriately selected patients. Studies have consistently demonstrated that patients undergoing aTSA experience substantial improvements in symptoms, mobility, and overall quality of life. This is particularly relevant for elderly patients, where preserving shoulder function and maintaining natural joint mechanics are key to achieving successful outcomes [6-9].

While reverse total shoulder arthroplasty (rTSA) has gained popularity in recent years, particularly for patients with rotator cuff arthropathy or severe cuff tears, it is not without its limitations [3]. rTSA is associated with unique risks, such as acromial and scapular spine fractures, inferior glenoid notching, and a potentially higher risk of deep infections compared to aTSA [10-13]. In contrast, aTSA is associated with superior restoration of shoulder motion, particularly in internal rotation, making it a more desirable treatment for most patients, especially with an intact rotator cuff [6,7,14-17]. Despite the growing trend toward rTSA in older patients, aTSA continues to demonstrate excellent outcomes in elderly patients with low rates of symptomatic rotator cuff failure and revision surgery [5-7].

Research on the outcomes of aTSA in elderly patients with intact rotator cuffs is limited. Our study addresses this gap by analysing functional results, outcomes, and revision rates associated with aTSA in this patient cohort. Focusing on patients over the age of 60 years with primary glenohumeral osteoarthritis and intact rotator cuffs, we examine the role of aTSA in this patient demographic and contribute further insights to aid surgical decision-making.

In this study, we report the outcomes of aTSA in a cohort of patients over 60 years old with intact rotator cuffs treated for glenohumeral arthritis, emphasising functional recovery, complication rates, and the need for revision surgery. However, given the limited outcome data on aTSA in elderly patients with an intact rotator cuff, this study aimed to quantify postoperative functional outcomes in patients aged 60 years and older using validated patient-reported outcome measures, including the OSS and the ASES scores.

## Materials and methods

Study design

This retrospective study included 42 patients aged 60 years and older who underwent anatomic total shoulder replacement. Patients were identified retrospectively using operative theatre logs and surgeon-maintained arthroplasty databases at two institutions. All arthroplasty procedures were performed by a single, fellowship-trained shoulder arthroplasty surgeon (DL), using a stemmed uncemented humeral component and keeled cemented polyethylene glenoid implant (Tornier Perform Anatomic Glenoid, Stryker Corporation, Kalamazoo, MI, USA), between 2019 and 2024. All patients provided informed consent for data collection.

The primary objective of this study was to assess the functional outcomes of aTSA in patients aged 60 years and older using validated patient-reported outcome measures. Functional outcomes were evaluated using the OSS and the ASES scores at baseline and at serial postoperative follow-up intervals. Secondary objectives included evaluating the extent of functional improvement following surgery and exploring the relationship between preoperative glenoid morphology, as classified by the Walch system, and postoperative outcomes.

Inclusion and exclusion criteria

Patient inclusion criteria were the following: (1) radiographically diagnosed severe shoulder arthritis accompanied by symptoms, (2) intact rotator cuff diagnosed either via ultrasound scan or MRI, (3) aged 60 and above, and (4) a minimum of 12 months follow-up period. Exclusion criteria included patients with a history of previous shoulder surgery, active infection, significant neurological deficits, those under the age of 60, and patients with less than one year of follow-up with OSS. Patients with incomplete preoperative or postoperative outcome data were excluded at the point of study selection.

Assessment and data collection

Demographics and clinical data were collected from the patient’s charts, theatre logs, and electronic medical records. Preoperative and postoperative OSS were collected from a combination of sources, including patient charts, electronic medical records, and clinic letters. Direct contact with patients via telephone or face-to-face consultation was also conducted to ensure accurate collection of postoperative OSS during follow-up.

The Walch classification is commonly used by surgeons when determining the treatment of osteoarthritis. However, its utility in prognosticating patients’ clinical state before and after TSA remains unproven. Preoperative CT scans were reviewed by a consultant surgeon, who classified the glenoid morphology in accordance with the system proposed by Walch et al. [17]. Patients with posterior humeral head subluxation exceeding 75% were excluded. Comparisons were performed to assess the ability of the Walch classification to predict the preoperative, postoperative, and improved state after TSA.

Functional outcomes were assessed using the OSS [1] and the ASES score [2]. Both scoring systems were utilised as validated and widely recognised measures for evaluating postoperative shoulder outcomes. Formal permission and a license to use the OSS were obtained from the University of Oxford. The ASES score is confirmed to be in the public domain, as verified by the American Shoulder and Elbow Surgeons (ASES), with an appropriate citation being sufficient for use. The OSS includes 12 items assessing shoulder pain and functional limitation, while the ASES score evaluates pain and activities of daily living. Together, these instruments provided a comprehensive measure of postoperative shoulder function, along with systematic documentation of complications and revision rates [1,2]. These multiple data sources enabled a comprehensive evaluation of the patients’ outcomes at baseline and at one, two, three, four, and five years following surgery.

Data for this study were collected as part of a quality improvement exercise, and the project was registered within our Surgical Specialties Division. Institutional oversight was provided by the Compliance and Clinical Effectiveness Facilitator, Patient Safety & Quality, Information Governance Department, Princess Alexandra Hospital (IBR: 4443).

Surgical technique

All anatomic shoulder arthroplasties were performed by a single surgeon across two sites using a standardised approach. Patients were assessed preoperatively by clinical examination, ultrasound assessment of rotator cuff integrity, and CT scan for templating using a three-dimensional planning software (Stryker Blueprint). All patients underwent stemmed aTSA via a deltopectoral approach for primary glenohumeral osteoarthritis. Patients were required to have a minimum follow-up of 12 months for inclusion.

The deltopectoral approach was used for all cases, and the subscapularis was detached from the lesser tuberosity by tenotomy and repaired with a transosseous suture on closure. The long head of the biceps underwent tenodesis if necessary. Both the proximal humerus and glenoid were prepared using standard operative technique for a stemmed uncemented humeral component and keeled cemented polyethylene glenoid implant.

The humeral head osteotomy was performed freehand following the removal of osteophytes and carefully executed along the anatomical neck, adhering to the patient’s natural retroversion to ensure anatomical accuracy. Sequential rasp insertion was then performed to size the humeral stem appropriately, while maintaining correct humeral retroversion throughout the process. After the glenoid was exposed and the labrum circumferentially resected, reaming was adjusted to the patient’s anatomy and glenoid morphology, as assessed from the preoperative three-dimensional template. Trial implants were inserted while stability, mobility, and soft tissue tension were assessed before definitive implant insertion. Soft tissue closure was performed in standard fashion, including transosseous subscapularis repair to the lesser trochanter.

Postoperative care and rehabilitation

All patients were discharged in a poly-sling limiting abduction and external rotation for six weeks. At the six-week mark, patients commenced rehabilitation under the guidance of the physiotherapy team. Follow-ups were scheduled at 2 and 6 weeks, and at 3, 6, 12 and 24 months postoperatively. Mobilisation at the wrist and elbow joint was encouraged from the immediate postoperative period, followed by progression to pendular exercises in week two and active-assisted or active range of movement in week six. Active strengthening, proprioception, and muscle patterning work was commenced at 10-12 weeks postoperatively. OSS and ASES scores were collected at six months, one year, and annually thereafter. Postoperative radiographs (anterior-posterior, axillary, and scapular Y views) were obtained at 6 weeks and 6 and 12 months and were prospectively reviewed by the operating surgeon.

Statistical analysis

Statistical analysis was performed using Microsoft Excel and Tableau software. Continuous variables are reported as mean ± standard deviation (SD) with ranges where applicable. Preoperative and postoperative OSS were compared using paired two-tailed t-tests. The mean difference, 95% confidence interval (CI), and p-values were calculated to assess the statistical significance of observed changes, with a threshold of p < 0.05. Only patients with complete outcome data were included in the analysis. Descriptive statistics were used to summarise patient demographics, glenoid morphology, and postoperative outcomes, providing an overview of the study cohort.

## Results

Demographic data

A total of 42 (100%) patients were included in the study, with a female predominance (n = 35, 83%) compared to males (n = 7, 16.7%). The patients had a mean age of 74.5 years (range = 62-89 years). The majority of patients were classified as American Society of Anesthesiologists (ASA) Physical Status Grade 2, indicating they had mild systemic disease, and all underwent stemmed conventional TSA for osteoarthritis. The mean follow-up duration was 2.5 years. Patient demographics and surgical characteristics are summarised in Table 1.

**Table 1:** Patient demographics and surgical characteristics.

Variable	Category	Number of patients (n)	Percentage (%)
Gender	Female	35	83.3
	Male	7	16.7
Mean age (years)	74.5 (65–89)	42	100
Type of glenoid (Walch)	A2	18	42.9
	A1	12	28.6
	B1	12	28.6
Surgical approach	Deltopectoral	42	100
Indication	Osteoarthritis	42	100
Mean follow-up (months)	30	42	100

Among the cases, the most common glenoid type was A2 (n = 18, 42.86%), characterised by mild posterior wear with concentric glenoid morphology, a typical pattern in glenohumeral osteoarthritis. B1 morphology was observed in 12 (28.57%) patients, reflecting significant posterior subluxation without severe erosion. Notably, over 75% posterior subluxation of the humeral head was not accepted, emphasising the importance of planning anatomical procedures based on detailed assessments. A1 glenoid type, indicative of minimal wear and earlier disease stages, was identified in 12 (28.57%) patients.

Preoperative and postoperative Oxford Shoulder Scores

The OSS was assessed for all 42 (100%) patients, with no cases lost to follow-up. Mean preoperative OSS was 19.02 (SD = 9.09), with scores ranging from 3 to 42, while the mean postoperative OSS improved significantly to 45.98 (SD = 2.9), with scores ranging from 38 to 48. A total of 40 (95.23%) patients achieved a postoperative OSS over 40. Notably, 20 (47.62%) patients attained the maximum score of 48, while 8 (19.5%) patients scored 46. Additionally,4 (9.52%) patients recorded scores of 45 and 47, with 3 (7.14%) patients achieving a score of 44. One (2.38%) patient achieved a score of 43. The analysis revealed a mean difference of 26.95 between preoperative and postoperative OSS, with a 95% confidence Interval for the mean change of 23.93 to 29.97, indicating a statistically significant improvement (p < 0.0001).

The American Shoulder and Elbow Surgeons score

**Figure 1:**
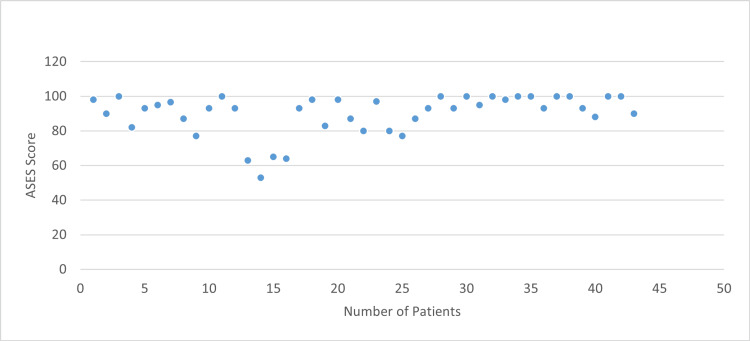
Distribution of American Shoulder and Elbow Surgeons (ASES) scores.

Additionally, functional outcomes were assessed postoperatively through the ASES scores. The data analysis revealed a mean ASES score of 90.06 among the 42 patients (SD = 11.59, range = 53-100). Most patients, 33 (78.57%), achieved postoperative ASES scores between 80 and 100. The distribution of postoperative assessment scores among patients is presented in Figure 1.

Patient-reported adverse outcomes

Postoperative adverse outcomes are summarised in Table 2. Among the 42 (100%) patients, the analysis of postoperative outcomes showed that 33 (78.57%) patients reported no complications or adverse symptoms following surgery. A total of 4 (9.52%) patients reported chronic postoperative pain, while another 4 (9.52%) experienced sensory changes or residual symptoms related to prominent pins following TSA. Additionally, 1 (2.38%) patient reported a restricted range of motion in the form of postoperative shoulder stiffness.

**Table 2:** Patient-reported adverse outcomes. The table summarises patient-reported adverse outcomes following anatomic total shoulder arthroplasty. No major complications were reported postoperatively. Percentages are expressed as n (%), representing the proportion of affected patients within the total cohort (n = 42).

Outcome type	Number of patients (n)	Percentage (%)
No adverse outcomes reported	33	78.57%
Postoperative chronic pain	4	9.52%
Occasional paraesthesia/sensory disturbance	4	9.52%
Restricted range of motion (stiffness)	1	2.38%

Revision cases

Our results indicated that 42 (100%) patients, including those who experienced postoperative adverse outcomes, successfully avoided the need for revision surgery or any additional surgical interventions at a mean of 2.5 years of follow-up.

Data summary

Table 3 consolidates functional outcomes, including pre and postoperative OSS, ASES scores, change in OSS, follow-up duration, and postoperative adverse events. Presenting these data in a structured format allows to quickly understand the magnitude of functional improvement and the incidence of complications within the cohort.

**Table 3:** Summary of preoperative and postoperative OSS and ASES scores. OSS = Oxford Shoulder Score; ASES = American Shoulder and Elbow Surgeons score

Variable	Mean	SD	Range
Preoperative OSS	19.02	9.09	3–42
Postoperative OSS	45.98	2.9	38–48
Change in OSS	26.95	-	-
ASES score	90.06	11.59	60–100

Postoperative functional outcomes, measured by the OSS, are presented in Table 4 according to age groups. The analysis of OSS across different age groups demonstrated a notable change in the mean following surgery. Patients aged 60-69 years had a mean preoperative OSS of 16, which increased to 45.4 postoperatively, resulting in a mean improvement of 29.4. In the 70-79-year age group, the mean preoperative OSS was 20.15, increasing to 46.6 postoperatively, with a mean improvement of 26.45. Patients aged 80-89 years showed a preoperative OSS of 16.37, which improved to 44.62 post-operatively, yielding a mean improvement of 28.25.

**Table 4:** Analysis of OSS across different age groups. OSS = Oxford Shoulder Score

Age group (years)	Mean preoperative OSS	Mean postoperative OSS	Mean improvement
65–69	16	45.4	29.4
70–79	20.15	46.6	26.45
80–89	16.37	44.62	28.25

## Discussion

Our study reveals significant clinical improvements for patients over 60 years old who received a primary aTSA, with many showing more than 100% improvement in OSS postoperatively. While there is a trend toward using reverse shoulder arthroplasty for older patients due to concerns about rotator cuff integrity, our findings suggest that aTSA is also effective in this age group. Patients in the 80-89-year age group showed a mean postoperative OSS improvement of 28.25, which was greater than the 70-79-year age group (26.45) but slightly lower than the 65-69-year age group (29.4). This suggests that older patients can experience a similar degree of functional improvement despite the greater physiological demands in younger individuals.

While reverse shoulder arthroplasty is often favoured in older patients due to concerns about secondary rotator cuff dysfunction potentially leading to implant failure [8,18,19], it is worth noting that we did not compare TSA to reverse shoulder arthroplasty in this study. However, many shoulder surgeons would typically opt for reverse shoulder arthroplasty for the aforementioned reasons, making it a significant consideration in surgical decision-making for elderly patients.

Analysis of shoulder arthroplasty database studies shows that elderly patients pose a higher risk of complications [20-23], which may be inherent to any major surgical procedure. Moreover, outcomes of failed anatomic shoulder replacements that are revised to reverse shoulder arthroplasty have found to be inferior to primary reverse shoulder arthroplasty [22]_._ This negates the argument that failed TSAs can later be revised to reverse shoulder arthroplasty and may prove a more difficult situation, both technically and physiologically, for the elderly patient.

Other studies have also reported excellent results in elderly patients with primary glenohumeral osteoarthritis and intact rotator cuffs. Jensen et al reported mean ASES scores of 78.3 and 76.7 for their cohort of 70-79 and 80+ year old patients, respectively. They observed a 0.8% revision rate in their study of 340 patients over a 3.3-year follow-up, aligning with the absence of revisions in our cohort. Furthermore, they found the incidence of secondary symptomatic rotator cuff tears in their population of TSA patients to be low.

Although existing research does not conclusively establish whether TSA or reverse shoulder arthroplasty is superior for achieving the best functional outcome, it clearly highlights the importance of selecting a procedure that minimises the likelihood of requiring revision surgery, especially in elderly patients.

Several other studies have compared reverse and anatomic shoulder arthroplasty in older patient groups [8,22,23]. Wright et al. compared 102 TSAs and 33 reverse shoulder arthroplasties for osteoarthritis with intact rotator cuff in patients over 70 years old. They reported no difference in pain, function, revisions or complications [8]. In another study by Triplet et al., the authors observed statistically significant improvement in ASES scores and patient satisfaction in the TSA group when compared to reverse shoulder arthroplasty patients older than 80 years at a minimum of two years of follow-up [24].

Further studies have reinforced the viability of anatomic TSA in patients over 80 years old, demonstrating favourable outcomes. Shimada et al. showed no significant difference in clinical outcomes or complication rates between Japanese patients aged 80 years or older and those 70 years or younger at mid-term follow-up [25,26]. Another study by Iriberri et al. regarding the use of aTSA in octogenarians reported good improvements in the range of movement and Constant scores [23]. In their analysis of 3,007 patients from a national database, Bovanratwet et al. suggested that TSA can be safely considered in octogenarians. They observed no significant difference in perioperative complications between patients over 80 years old compared to 70-79 year olds [27,28]. However, the study noted higher readmission rates for pneumonia and urinary tract infections among octogenarians, as well as increased length of stay compared to patients under 70 years. These findings underscore the necessity of careful patient selection and the consideration of comorbidities when planning shoulder arthroplasty in older adults.

The perceived increased risk of secondary rotator cuff dysfunction is considered by many as a contraindication for anatomic TSA. In their five-year follow-up of TSA patients, Young et al. found a 16.8% rate of secondary rotator cuff dysfunction identified by superior subluxation of the humeral head on radiographs [19]. Notably, of their 518 patients, only one required revision to reverse shoulder arthroplasty, suggesting that many maintain a satisfactory level of clinical function despite possible cuff deficiency. In concordance, Poondla et al. observed a low revision rate of 1% for rotator cuff dysfunction among 482 TSA patients, with a higher total revision rate among reverse shoulder arthroplasty patients in early to mid-term follow-up [11]. These findings align with our research, suggesting that TSA can offer a reasonable level of function for elderly patients, even with potential secondary rotator cuff insufficiency.

Strengths of this study include clearly defined inclusion and exclusion criteria, a consecutive patient cohort, and careful preoperative patient selection supported by CT imaging and standardised Walch glenoid classification. All procedures were performed by a single experienced shoulder arthroplasty surgeon using a standardised surgical technique and postoperative follow-up protocol, ensuring procedural consistency. Functional outcomes were assessed using two validated and widely used patient-reported outcome measures, i.e., the OSS and the ASES score, which are recognised nationally and internationally. Complete follow-up data were available for all patients. Collectively, these factors enhance the internal validity of the study and allow a focused evaluation of aTSA outcomes in an elderly rotator cuff-intact population.

Limitations

This study has several limitations that must be acknowledged. First, it is a retrospective, single-surgeon series with standardised implant selection, surgical technique, and follow-up protocols. While this ensures consistency, it may introduce surgeon expertise bias and limit generalisability to lower-volume centres. Functional outcomes may also have been influenced by institution-specific postoperative rehabilitation pathways, and therefore, the findings are most applicable to carefully selected elderly patients managed in specialised centres.

Second, the maximum follow-up for our study was limited to five years, which may not fully capture long-term complications and outcomes. Furthermore, revision rates represent a key limitation of this study. With a mean follow-up of 30 months and no revisions observed among the 42 patients included over a five-year period, the study is underpowered to assess revision risk. Future research with larger cohorts and longer follow-up is needed to determine the true need for revision after aTSA in elderly rotator-cuff-intact patients.

Third, the study included only eight patients aged 80 or older, which limits the statistical power to detect differences between younger and older patient groups. Finally, reverse shoulder arthroplasty patients were not included for comparison, which prevents us from drawing definitive conclusions about the relative suitability of TSA versus reverse shoulder arthroplasty in the elderly population.

## Conclusions

aTSA may be considered for elderly patients with glenohumeral arthritis and an intact rotator cuff. In this retrospective cohort, we demonstrated comparable outcomes among patients in their 60s, 70s, and 80s with no major complications or revisions at early to mid-term follow-up. While some concerns regarding secondary rotator cuff tears in the elderly population receiving a TSA may be justified, our findings suggest that satisfactory clinical function can be achieved in this age group. The population is expected to live longer, and therefore, both longevity of the chosen implant and risk to the patient must be primary considerations. Further prospective studies are needed to determine whether TSA is the optimal implant choice for elderly patients with intact rotator cuff, and to clarify the role of reverse shoulder arthroplasty in primary glenohumeral osteoarthritis.
